# Decisions by regulatory agencies: are they evidence-based?

**DOI:** 10.1186/1745-6215-8-13

**Published:** 2007-04-11

**Authors:** Curt D Furberg

**Affiliations:** 1 Division of Public Health Sciences, Wake Forest University School of Medicine, Medical Center Boulevard, Winston-Salem, North Carolina 27157-1063, USA

## Abstract

Contradictory statements about the non-steroidal anti-inflammatory drugs from the European Medicines Agency and the United States Food and Drug Administration have raised questions about whether regulatory decisions are evidence-based. For the selective COX-2 inhibitors, there are clear contraindications and warnings in Europe, but only a vaguely worded Black Box warning in the United States. All the non-selective agents are given an almost "clean bill of health" in Europe, while all of them are judged to have a similar risk-benefit ratio as celecoxib in the United States. The regulatory agencies have failed to recognize the clinical trial evidence that the risk of cardiovascular events varies substantially among the non-selective agents, with diclofenac carrying the highest risk of harm.

## Background

Decisions by regulatory agencies follow explicit regulations and should be evidence-based. An established practice has been that approval of a new drug requires two independent clinical trials documenting safety and efficacy for the drug's intended use. But are the regulatory agencies rigorously ensuring that decisions are evidence-based? Contradictory statements about the non-steroidal anti-inflammatory drugs (NSAIDs) from the European Medicines Agency (EMEA) and the U.S. Food and Drug Administration (FDA) have raised this question.

## Discussion

### Selective COX-2 inhibitors

An FDA Advisory Committee convened in February, 2005 to review primarily the three selective COX-2 inhibitors available in the U.S. It concluded overwhelmingly (32 votes to zero) that these agents increase the risk of thrombotic cardiovascular events[1]. The evidence from several placebo-controlled clinical trials was considered conclusive. The problem was interpreted as a class effect, although the degree of harm appeared to differ among the agents. The Advisory Committee recommended that celecoxib remain on the market with major restrictions applied to its use[1].

EMEA was in agreement with the FDA and recommended suspension of valdecoxib[2]. It also added new contraindications and warnings to the other marketed coxibs. Contraindications were added for patients with established ischemic vascular disease and reinforced warnings were issued for patients with risk factors of heart disease. Based on the same available scientific evidence, the FDA did not follow the recommendations by its Advisory Committee[3]. Rather the FDA added only a Black Box warning vaguely stating that celecoxib "*may *(author's emphasis) cause an increased risk of serious cardiovascular events,..." and that "Patients with cardiovascular disease or risk factors for cardiovascular disease *may *(author's emphasis) be at greater risk"[4].

### Non-selective NSAIDs

The recommendations for the non-selective NSAIDs by FDA and EMEA issued in 2006 also went in a different direction. EMEA concluded that the risk-benefit balance for eleven of these agents remains favorable[5]. However, it could not exclude "a small increase in risk of thrombotic events." FDA added to the Black Box warning for celecoxib that "All NSAIDs *may *(author's emphasis) have a similar risk. This risk *may *(author's emphasis) increase with duration of use"[4]. Again, the different conclusions by the regulatory agencies were based on the same available scientific evidence.

So what is the evidence for cardiotoxic effects of non-selective NSAIDs? The safety information is limited, with no large, long-term, placebo-controlled trials. In their meta-analysis, Kearney et al.[6] reported summary rate ratios for high doses of naproxen, ibuprofen and diclofenac in comparison with placebo; these ratios were 0.92 (95% CI 0.67 to1.26), 1.51 (0.96 to 2.37) and 1.63 (1.12 to 2.37), respectively. The authors concluded "Our results indicated that high-dose ibuprofen (800 mg three times daily) and high-dose diclofenac (75 mg twice daily) were each associated with an increased risk of vascular events, but that the risks of high-dose naproxen (500 mg twice daily) were substantially smaller."

A recent indirect comparison supports these findings[7]. In 26 active-control trials comparing COX-2 inhibitors to diclofenac, the risk of vascular events was lower with the COX-2 inhibitors (relative risk 0.92; 95% CI, 0.81–1.05). For trials comparing COX-2 inhibitors to naproxen, the former were associated with an increased vascular risk (relative risk 1.57; 95% CI, 1.21 to 2.03). Thus, compared to naproxen, diclofenac may increase the vascular risk by about 70%[7].

The scientific evidence points to major differences among the non-selective NSAIDs. Naproxen appears to be fairly neutral in its cardiovascular effects. In fact, at the FDA Hearing in February 2005, the Advisory Committee recommended that naproxen be the preferred NSAID comparator in future trials of painkillers[1]. Diclofenac has pharmacologic effects similar to those of celecoxib. The evidence is fairly overwhelming that this drug increases the risk of cardiovascular events. However, the regulatory agencies so far have not recognized these clinically important differences among the non-selective NSAIDs. Since diclofenac is the most commonly used non-selective NSAID and since it increases the risk of vascular events by 60–70%, the unrecognized harm it has caused worldwide could be enormous. Consideration ought to be given to removing this drug from the market, since there is not evidence that it offers better pain control than the large number of non-selective NSAIDs on the market.

## Conclusion

EMEA gave all non-selective NSAIDs an almost "clean bill of health" while the FDA created an unnecessary "health" scare among patients using these drugs by suggesting that all of them have vascular risks similar to celecoxib. Both positions are partially right according to the scientific evidence, since some non-selective NSAIDs increase risk of cardiovascular events, while others do not. Being half-right is not good enough. The time has come for EMEA and FDA to set the record straight, based on current scientific evidence.
